# Asafœtida a Preventive of Measles

**Published:** 1845-05

**Authors:** 


### ASAFŒTIDA A PREVENTIVE OF MEASLES.

So Dr. R. B. Harper, of Bloomington, Tennessee, thinks he has discovered, and the facts upon which rests his belief we give below. The first question that will arise is, do the cases cited make out the point—is it true? That is a very essential question which must first be settled in all such cases. The inquiry would naturally afterwards come up, what is the *modus operandi* of the article? Is it homœopathic? Does asafœtida cause measles in the healthy subject? If not, one of two things must be true;—either that Hahnemann missed it, in this instance, or else asafœtida is not a preventive of measles. We shall not discuss any of these subordinate questions until the primary one is put to rest, and in order that other observers may turn their eyes towards the matter, we publish Dr. Harper's cases as they were lately communicated to us. Y.

"In the fall of 1842, Rubeola prevailed in this region of country in a form unusually aggravated. Reasons existed with me why I should be peculiarly anxious to keep the disease out of my family, none of whom had ever had measles, and as asafœtida had been mentioned as possessed of prophylactic virtues, I resolved on giving that article a trial. Accordingly I made a saturated tincture of it, of which I took a table-spoonful three times a day, at the same time carrying a lump of asafœtida in my pocket, and swallowing small pieces of it occasionally. I was particular to take it in some of these forms just before visiting a case of measles. My family also made free use of the remedy. Among others, I attended the following cases during which time I was exposed to the disease:

"1. I was called, October 11th, to see Mrs. D. whom I found covered with the eruption of measles, and suffering from high fever, nausea and vomiting. In a minute examination which I made of all the features of the case I was fully exposed to the infection, and having up to this time been quite susceptible to contagious affections, I supposed that I had the best chances to take measles from my patient. But nothing of the sort occurred.

"2. October 16th, I was called to visit the child of Mr. J. M. with measles; I remained in the house during the night following, and examined a case of measles in a negro, in addition to the case of the child.

"3. Oct. 20th, I visited Mrs. W. with measles, and found her also in labor. The eruption was fully established, and was attended with high febrile excitement, but notwithstanding these unpleasant circumstances, the labor progressed favorably, and in a short time she was delivered of a well grown child, and healthy in every respect, except that it was covered with measles. I was necessarily much about the patient during her labor, but nevertheless escaped her disease, and thus passed through the epidemic without contracting it. The mother and child in this case both did well.

"I might speculate on these facts, but will leave that task to others. If my experience of the powers of asafœtida in this respect should be confirmed, the discovery will be valuable. The point, however, requires additional investigation. In medicine, everything depends upon experience. Accident has placed in our hands many of our most precious remedies, which, probably, when first proposed, commanded but slight attention, and awakened, if not violent opposition, or merciless ridicule, at best only feeble expectations. But bark, mercury, iodine, and the vaccine virus are agents the potency of which in their appropriate maladies, no one now questions, although no one professes to be able to explain their mode of curing or preventing disease."

